# Rice *KORPOKKUR* gene is expressed in mitotic cells and regulates pleiotropic features during vegetative phase

**DOI:** 10.5511/plantbiotechnology.24.0305a

**Published:** 2024-06-25

**Authors:** Kaito Chiba, Takumi Tezuka, Hiroetsu Wabiko, Yasuo Nagato, Nobuhiro Nagasawa, Namiko Satoh-Nagasawa

**Affiliations:** 1 Department of Biological Production, Faculty of Bioresource Sciences, Akita Prefectural University; 2 National Institute of Genetics; 3 Graduate School of Agricultural and Life Sciences, The University of Tokyo

**Keywords:** cell division, cytokinesis, phase change

## Abstract

Cell division is important for organisms to grow and repair damaged tissues. A mutant screen in rice has identified dwarf *korpokkur* (*kor*) mutants that code for a novel protein potentially involved in mitosis including cytokinesis in rice. The *KOR* gene is expressed during the mitotic phase and a defect in the *KOR* gene induces cells with two nuclei. Analysis of *kor* mutants suggests that the *KOR* gene promotes cell division in the rice leaf primordia for a period after initiation, and maintains proper cell morphology especially in non-meristematic tissues. Additionally, *kor* mutants showed a delayed transition from juvenile phase to adult phase. Future research will shed light on the relationship between the mitotic defect and other features observed in the *kor* mutants.

## Introduction

Cell division is a fundamental event for life that enables organisms to grow and repair damaged tissues. The series of events that take place within a cell leading to its division into two daughter cells is termed cell cycle. The two phases of the cell cycle are the interphase and the mitotic phase (M phase). During the cell cycle, DNA replicates accurately ensuring equal distribution of DNA into each daughter cell, thereby maintaining genome stability. DNA replication takes place during the synthetic phase (S phase) in the interphase and the distribution of DNA is completed by cytokinesis, the final step of the M phase.

Cell division is accomplished by the highly dynamic cytoskeleton. While animals and yeast rely on the actin cytoskeleton, plants have evolved unique mechanisms that utilize the microtubule cytoskeleton. For example, the preprophase band (PPB) and the phragmoplast (PHP) are plant-specific structures. PPB is formed at the end of interphase and the beginning of M phase by selective microtubule stabilization (Lipka et al. 2015). It outlines the periphery of the future division plane. In contrast, PHP functions at the end of the M phase. Many membrane vesicles containing cell plate components are delivered along the PHP and the cell plate expands laterally (Sinclair et al. 2022; Smertenko et al. 2017). Many proteins involved in the construction and function of PPB and PHP have been identified, particularly in *Arabidopsis* (Lipka et al. 2015). For example, the protein phosphatase complex that includes TONNEAU1 (TON1), TON1 RECRUITING MOTIF and protein phosphatase 2 A, is essential for the formation of the PPB (Camilleri et al. 2002; Spinner et al. 2013). On the other hand, mutant screens in *Arabidopsis* have also identified a cytokinesis-specific syntaxin named KNOLLE and an interacting Sec1/Munc18 protein named KEULE; both proteins are required for vesicle fusion in PHP during cytokinesis (Assaad et al. 2001; Otegui et al. 2005; Park et al. 2023, 2012; Söllner et al. 2002). Despite numerous studies aimed at elucidating the mechanisms of cell division many questions remain to be answered.

Here, we present an analysis of rice *korpokkur* (*kor*) mutants exhibiting a dwarf phenotype. Map-based cloning revealed that *KOR* encodes a novel protein presumed to be involved in mitosis including cytokinesis by cell morphology observations of the *kor* mutants and analysis of *KOR* gene expression by in situ hybridization.

## Materials and methods

### Plant materials

Two allelic single-gene recessive mutants of rice were identified in M_2_ populations of the japonica variety Akitakomachi mutagenized with N-methyl-N-nitrosourea. Plants were grown on Murashige–Skoog medium at 28°C under continuous light conditions.

### Paraffin sectioning and histological analysis

Plant samples from *kor* and wild-type were fixed with 4% (w/v) paraformaldehyde and 1% Triton-X in 0.1 M sodium phosphate buffer for 48 h at 4°C. Samples were then dehydrated in a gradual ethanol series, substituted with t-butanol, and embedded in Paraplast Plus. Sectioning was performed at a thickness of 10 µm using a rotary microtome. The sections were stained with haematoxylin for histological analysis or with DAPI for nuclear detection.

### Epidermal cell observations

The 3rd-leaf blades of *kor* and wild-type plants were fixed as described above and dehydrated using a graded ethanol series. Dehydrated samples were incubated at 96°C in chloral hydrate dissolved in 100% ethanol until they cleared and were then observed under a light microscope.

### Real-time PCR analysis

Real-time PCR was performed using TaqMan MicroRNA assay (Applied Biosystems). RNAs were isolated using the TRIzol reagent (Invitrogen) from the second, the fifth and the eighth leaves of *kor-1* and the second and fifth leaves of wild-type. The eighth leaf of *kor-1* and fifth leaf of wild-type expanded at the same time. We used the *Oryza sativa* *UBIQITIN5* (*OsUBQ5*) gene as an internal control, and quantitative PCR was conducted using KOD SYBR qPCR Mix (TOYOBO). The primers used for *OsUBQ5* were as follows: forward primer (5' ACCACTTCGACCGCCACTACT 3') and reverse primer (5' ACGCCTAAGCCTGCTGGTT 3').

### Flow cytometric analysis

The 5th leaves of *kor-1* and wild-type were cut into small pieces in 430 µl of extraction buffer (CyStain UV Precise P), filtered through a 20 µm mesh, and mixed with 1.6 ml of nuclear staining buffer (Sysmex Europe SE). The samples were analyzed with a CyFlow space (Sysmex Europe SE).

### Map-based cloning

*KOR* gene, *kor-1* heterozygote plants were crossed with the indica variety Kasalath, and F_2_ plants showing the *kor* mutant phenotype were used for mapping.

### In situ hybridization

For studying *KOR* expression patterns by in situ hybridization, a full-length cDNA clone (J033038D16) digested with *Eco*R I was transcribed to generate RNA probes. Digoxygenin-labelled antisense and sense RNA probes were prepared. Single target in situ hybridization was performed following the methods described in Kouchi and Hata (1993). For double target in situ hybridization, digoxygenin-labelled and biotin-labelled probes for *KOR* and *CycOs2* (Os08g0512600), fluorescein-labelled and digoxygenin-labelled probes for *KOR* and *Histone H4*, respectively, were used. Hybridization of probes, post-hybridization washes, and blocking procedures were performed according to the methods of Kouchi and Hata (1993). For double target in situ hybridization for *KOR* and *CycOs2*, an HNPP Fluorescent Detection Set (Roche) was used for the detection of digoxygenin-labelled probe and a TSA™ Biotin System (PerkinElmer) together with streptavidin-fluorescein (PerkinElmer) was used for the detection of biotin-labelled probes following the manufacturer’s protocol. The slides were washed in sterile distilled water and mounted with Prolong Gold Antifade Reagent containing DAPI (Invitrogen). They were observed with a fluorescence microscope using B excitation band pass, G excitation band pass and DAPI band pass filters for fluorescein, HNP/TR, and DAPI signals, respectively. For double target in situ hybridization for *KOR* and *Histone H4*, a NBT/BCIP system was used for the detection of fluorescein-labelled probe and a Fast Red system was used for the detection of digoxygenin-labelled probes.

## Results and discussion

**Figure 1:**
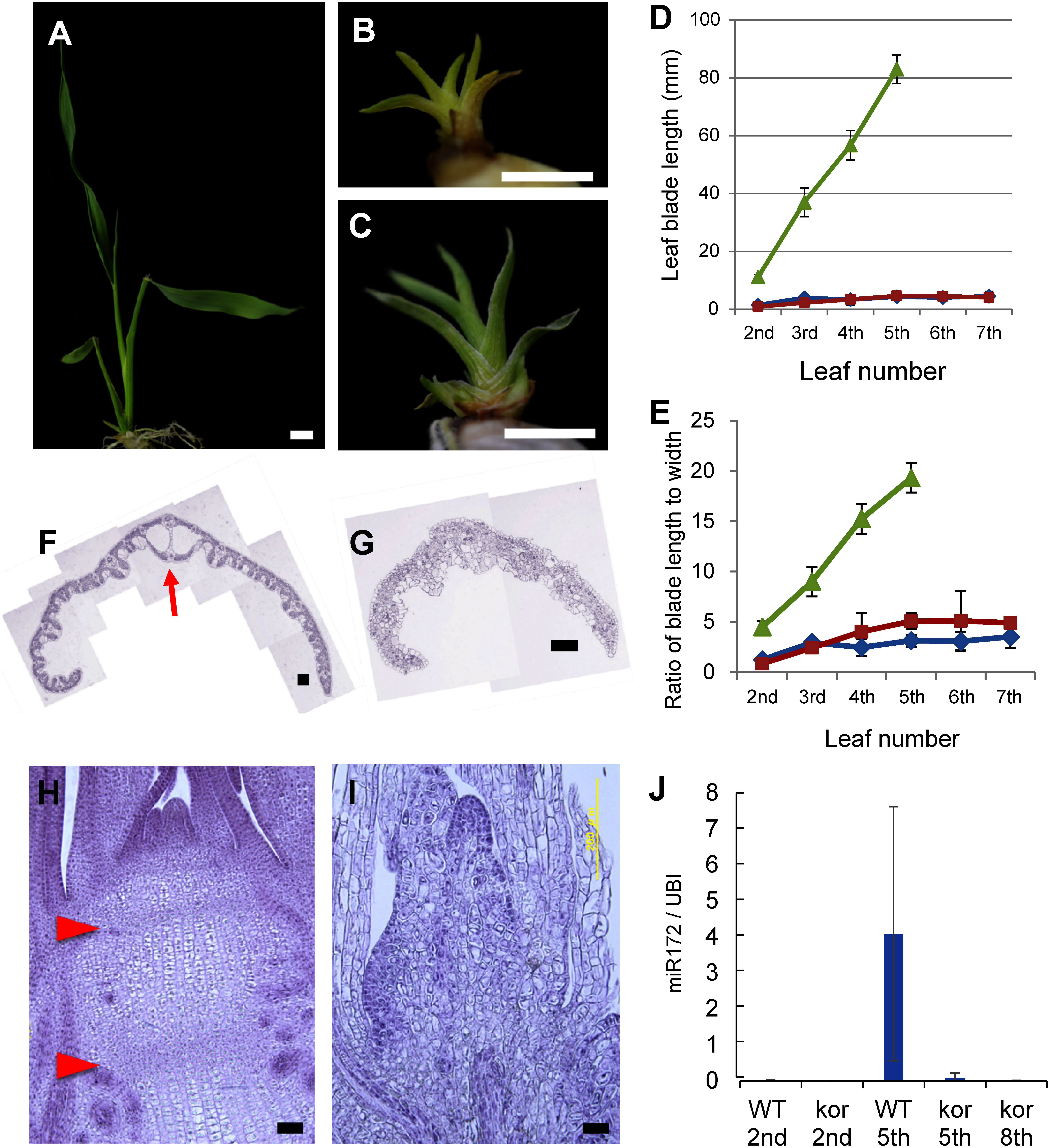
Figure 1. *kor* mutants exhibit a dwarf phenotype and prolonged juvenile phase. A–C. 10 days-old seedlings of wild type (A), *kor-1* (B) and *kor-2* (C). D. Leaf blade length in wild-type (green line), *kor-1* (blue line) and *kor-2* (red line). E. Ratio of leaf blade length to leaf blade width in wild-type (green line), *kor-1* (blue line) and *kor-2* (red line). F, G. Cross sections of wild type (F) and *kor-1* (G) 5th leaf blade cut at half-length from the base. Red arrow shows midrib structure. H, I. Cross section of 3-weeks-old stem structure of wild type (H) and *kor-1* (I) plants. Red arrowheads indicate position of nodes. J. Real-time PCR analysis of *miR172* expression in wild-type and *kor-1* mutant leaf blades. Data represent mean±SD in D, E and J (*n*=3). Bars=5 mm in A to C, 100 µm in F, G and 50 µm in H and I. A, F, H: wild-type. B, G, I: *kor-1*. C: *kor-2*.

We identified two recessive rice mutants, shown to be allelic, exhibiting a dwarf phenotype (Figure 1A, B, C). The mutants continuously produced markedly undersized leaves without progressing to the flowering stage. Because of their dwarf appearance, we named them *korpokkur* (*kor*) in reference to the dwarf-like mythological creatures in folklore of the Ainu people of the northern Japanese islands. More than half of the mutant plants lost their ability to perform cell division and died during vegetative development. However, a number of mutants grew further as described below.

In the wild type, the leaf blade length increases as the leaf position elevates. In contrast, the leaf blades of *kor* mutants stay short (Figure 1D). Additionally, while the ratio of leaf blade length to width increases in the wild type, no significant changes were observed in the *kor* mutants (Figure 1E). To further compare leaf anatomy between mutant and wild type we made observations of cross sections of the 5th leaf blade. In the wild type, the midrib was observed at half leaf-length from the base (Figure 1F). However, this structure was not observed in the 5th leaves of *kor* mutants (Figure 1G). We also investigated longitudinal sections of the stem structure and observed nodes and internodes in 3-weeks-old wild-type plants (Figure 1H). However, these structures were not obvious in *kor* mutants (Figure 1I). Because *kor* mutants resemble the heterochronic *mori1* mutant, which is affected in the juvenile-to-adult phase transition (Asai et al. 2002), we suspected that the transition from juvenile to adult phase may also be delayed in the *kor* mutants. We performed real-time PCR analyses with *miR172*, a miRNA which acts downstream of *miR156*, a key regulator of juvenile to adult transition in plants. *miR172* expression is known to increase during the phase transition. Indeed, *miR172* expression levels in the 5th and even 8th leaves in *kor* mutants were lower than in the 5th leaves in wild type (Figure 1J). These results indicate that *KOR* is necessary for the juvenile-to-adult phase change in rice.

**Figure 2:**
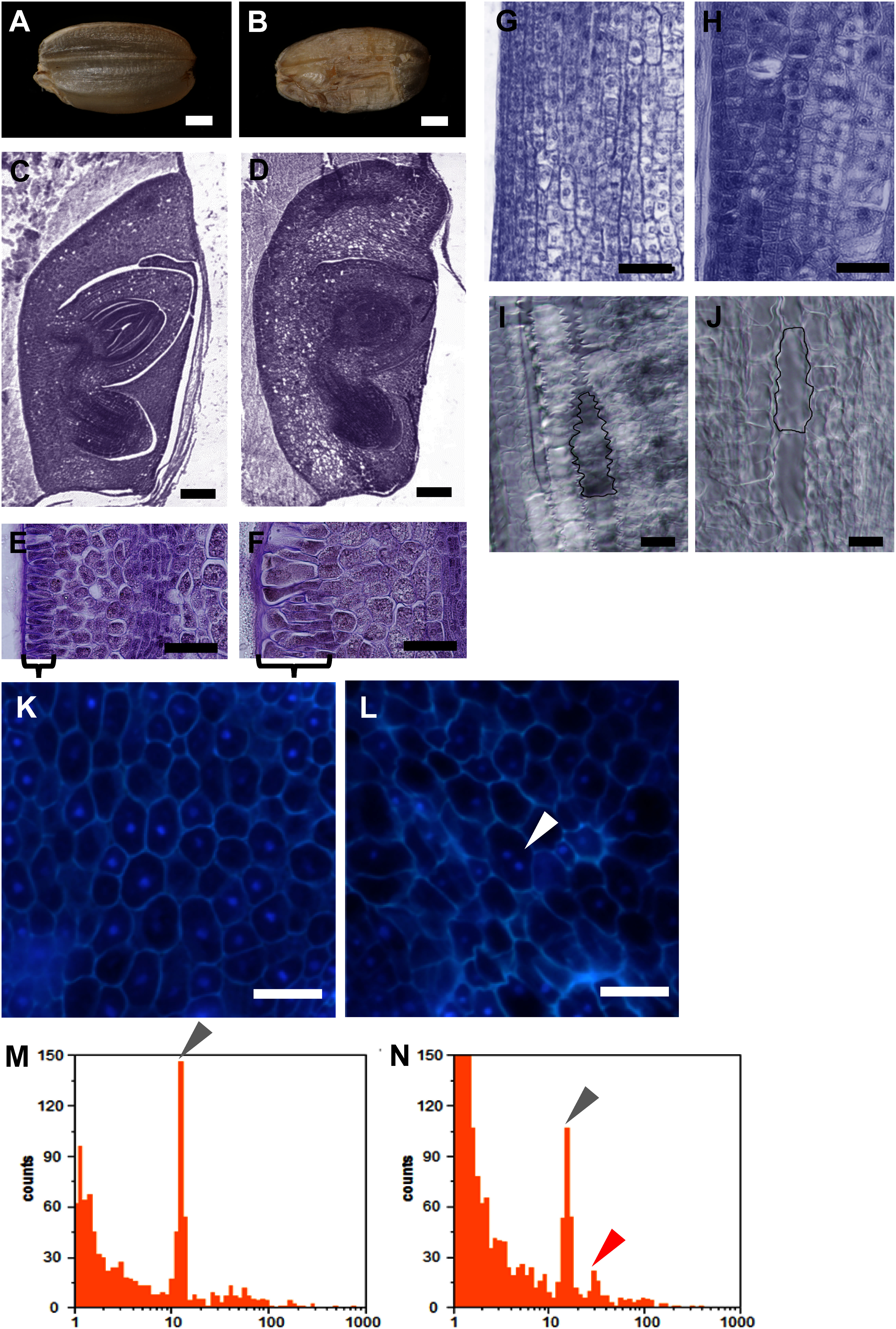
Figure 2. *kor* mutants have aberrant morphology and cells with two nuclei. A, B. Mature seeds. Bars: 1 mm in A, B. C, D. Mature embryos. E, F. Epidermal cells in rice scutellum. Black brackets indicate the outer most layer of the scutellum. G, H. Cells in rice leaf sheath. I, J. Epidermal cells in rice leaf blades. The representative cells are outlined in black. K, L. Cells in rice scutellum stained with DAPI. White arrowhead indicates a cell with two nuclei. M, N. Flow cytometry analysis of DNA content in mature leaf-blade cells of wild-type and *kor-1* plants. Gray arrowheads show peaks of 2C cells and red arrowhead shows a peak of 4C cells. A, C, E, G, I, K: wild-type. B, D, F, H, J, L: *kor-1*. Bars: 250 µm in C, D, 50 µm in E to H and K, L, 10 µm in I, J.

However, the *kor* mutant is distinct, particularly in cell morphology, from other mutants such as *peterpan syndrome* and *precocious*, which are defective in the timing of the juvenile-to-adult phase change (Hibara et al. 2016; Tanaka et al. 2011). First, we could distinguish *kor* mutant seeds by their wrinkled endosperm (Figure 2A, B). *kor* mutant mature seeds contain small starch granules whose sizes were similar to the one of 10 days-after-pollinated wild type seeds. Undeveloped starch granules can be one of the reasons of wrinkled endosperm in *kor* mutant. Next, the overall shape of embryos, especially the external shape of the scutellum of *kor* mutants was bulky (Figure 2C, D). In addition, the arrangement of cells in the outermost layer of the scutellum was irregular (Figure 2E, F). The cell shape and cell size in the mature leaf sheath of *kor* mutant were also variable (Figure 2G, H). Thus, cells of several non-meristematic organ in the *kor* mutant showed irregular shapes suggesting that *KOR* is essential for proper cell shape in rice. Although the size of the seeds and embryos of *kor* mutants are indistinguishable from wild type, the size of the leaves in *kor* mutants was noticeably smaller, as described above. To determine whether the size of the cells or their number was responsible for the small size of whole leaves, we observed leaf blade epidermal cells. As shown in Figure 2I and 2J the size of the cells was not significantly different between wild-type and *kor* mutants, however the wavy shape of the cell outline was different. The length of the waves is shorter in wild-type. This indicated that one of the reasons why *kor* mutants display a dwarf phenotype is a reduced number of cells. This suggests that the frequency of cell division in the *kor* mutant is reduced compared to wild type. Thus, KOR’s function is to promote cell division, at least in some organs, in rice. More detailed observations of the *kor* mutant scutellum revealed the existence of a non-negligible number of cells with two nuclei (Figure 2K, L). This suggests that the cytokinesis of cells in *kor* mutant is incomplete. To verify the status of DNA content of the nuclei, we performed flow-cytometry analysis of mature leaf blade cells. Since most of wild-type leaf blade cells are not undergoing cell division, only one peak of cells with 2C-DNA (diploid) was visible. In contrast, two peaks of cells, a larger one with 2C-DNA (diploid) and smaller one with 4C-DNA (tetraploid) were observed in the *kor* mutant (Figure 2M, N). These results suggest that mitosis including cytokinesis is abnormal in some cells of the *kor* mutant.

**Figure 3:**
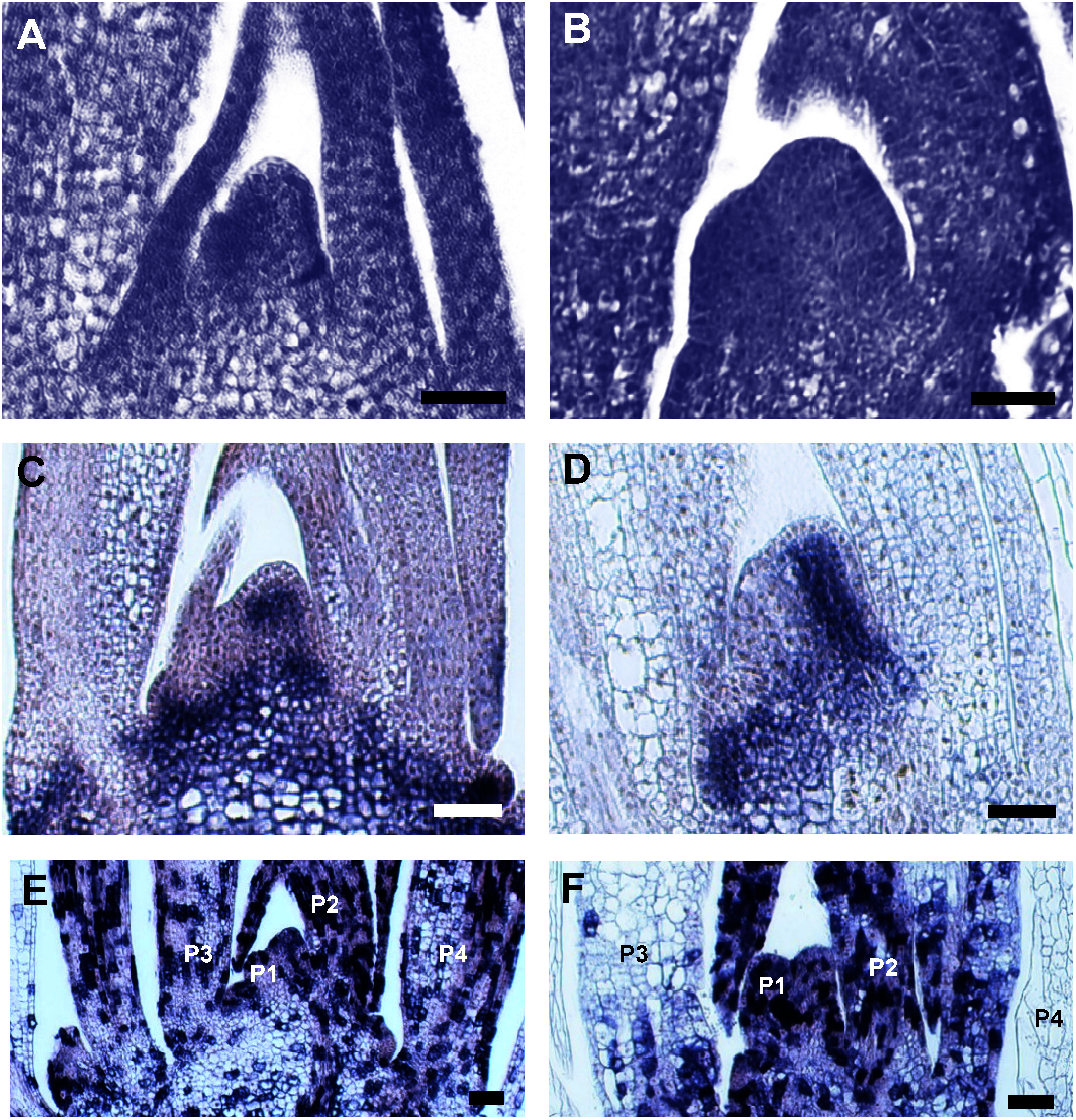
Figure 3. SAM morphology and gene expressions in wild-type and *kor* mutants. A, B. Morphology of SAM. C, D. *OSH1* expression patterns in SAM. E, F. *Histone H4* expression patterns in SAM. A, C, E: wild-type. B, D, F: *kor* mutants. Bars: 50 µm.

Interestingly, no major defects of cell morphology around SAM and RAM in *kor* embryos were observed (Figure 2C, D). Similarly, after germination the shape and array of cells in the SAM appear to be normal in the *kor* mutant. However, the SAM in *kor* mutants were flatter than the ones in the wild-type and young leaf primordia looked slightly bigger than wild-type’s (Figure 3A, B). In order to get insights into the organization of the SAM, we performed in situ hybridization using molecular marker genes for the SAM. The expression of *OSH1*, which is expressed in the undifferentiated cells around the SAM, was observed in both the mutant and the wild-type (Figure 3C, D). As for *Histone H4* which expression is induced at the G1/S transition followed by quick decay of transcripts at the end of the S phase (Reichheld et al. 1998), there was no significant differences in the SAM, P1 and P2 signals in leaf primordia between wild-type and *kor* mutants. However, the signals were absent in *kor* mutants in the cells of P3 and P4 leaf primordia where multiple signals were observed in wild type (Figure 3E, F).

Based on the results of in situ hybridizations with the molecular markers, the maintenance of undifferentiated cells and cell division frequency in the SAM were relatively normal in *kor* mutants. On the other hand, we observed that the P3 and P4 leaves in the *kor* mutants have a quite lower cell division frequency than in the wild type. This suggests that *KOR* is responsible for cell division activity in several leaves after initiation in rice.

**Figure 4:**
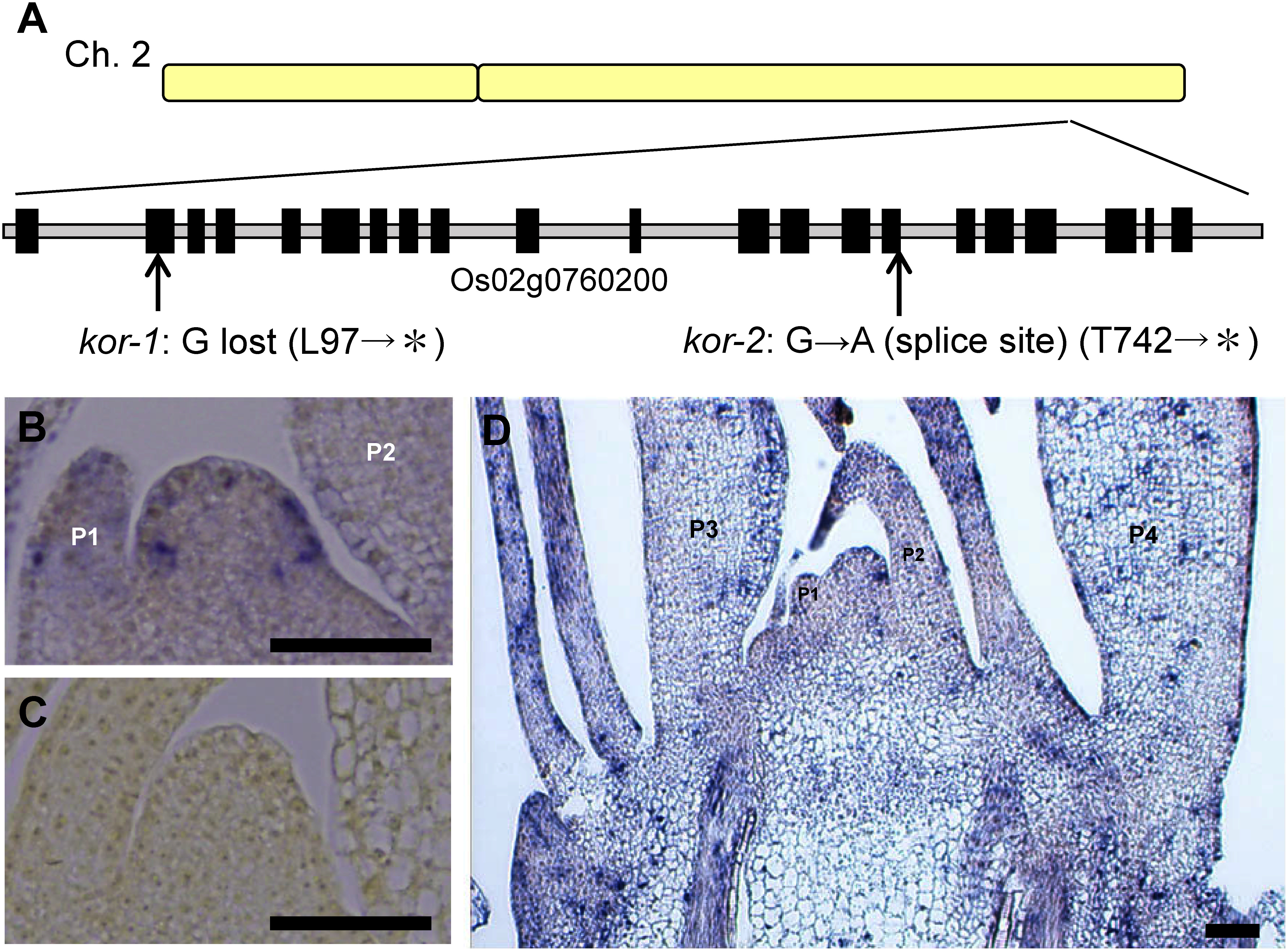
Figure 4. *KOR* gene structure and expression pattern in rice shoot apex. A: *KOR* gene structure showing the position of the *kor-1* and *kor-2* mutations. B and C: *KOR* expression patterns in SAM with antisense (B) and sense (C) probe. D: *KOR* expression pattern in shoot apex. Bars: 50 µm.

Rough mapping of F_2_ plants from *kor-1* and Kasalath (ssp *indica*) with pooled DNA revealed that the *KOR* locus maps on the long arm of chromosome 2. Finer mapping placed the *KOR* locus between 31,985,954 bp and 32,086,063 bp. Among the 22 genes in this region, one annotated plant-specific gene, Os02g0760200, has a point mutation in an exon. The *kor-1* mutation consists of a single-base deletion (G (229)) in the second exon of the gene, generating a premature stop codon in the same gene. The *kor-2* mutation consists of a single base change (G to A) at the splicing acceptor site of the 15th intron of the gene and the mutation also caused a premature stop codon (Figure 4A). These results indicate that Os02g0760200 is the causal gene of the *kor* mutation. While annotated as a protein containing a C2 calcium/lipid-binding region, a CaLB domain, as well as a predicted 3d structure similar to the CaLB domain, homology at the amino acid level is quite low, making it challenging to definitively state that KOR has CaLB domain.

In order to conjecture the function of this novel protein, we performed expression analysis. Semi-quantitative RT-PCR showed that *KOR* is expressed strongly around meristems and broadly in whole rice plants (Supplementary Figure S1). Interestingly, the expression pattern of *KOR* was patchy (Figure 4B, D). The signal was found mainly in P1 to P4 leaf primordia. The patchy expression pattern suggests that *KOR* expression is associated with the cell cycle.

**Figure 5:**
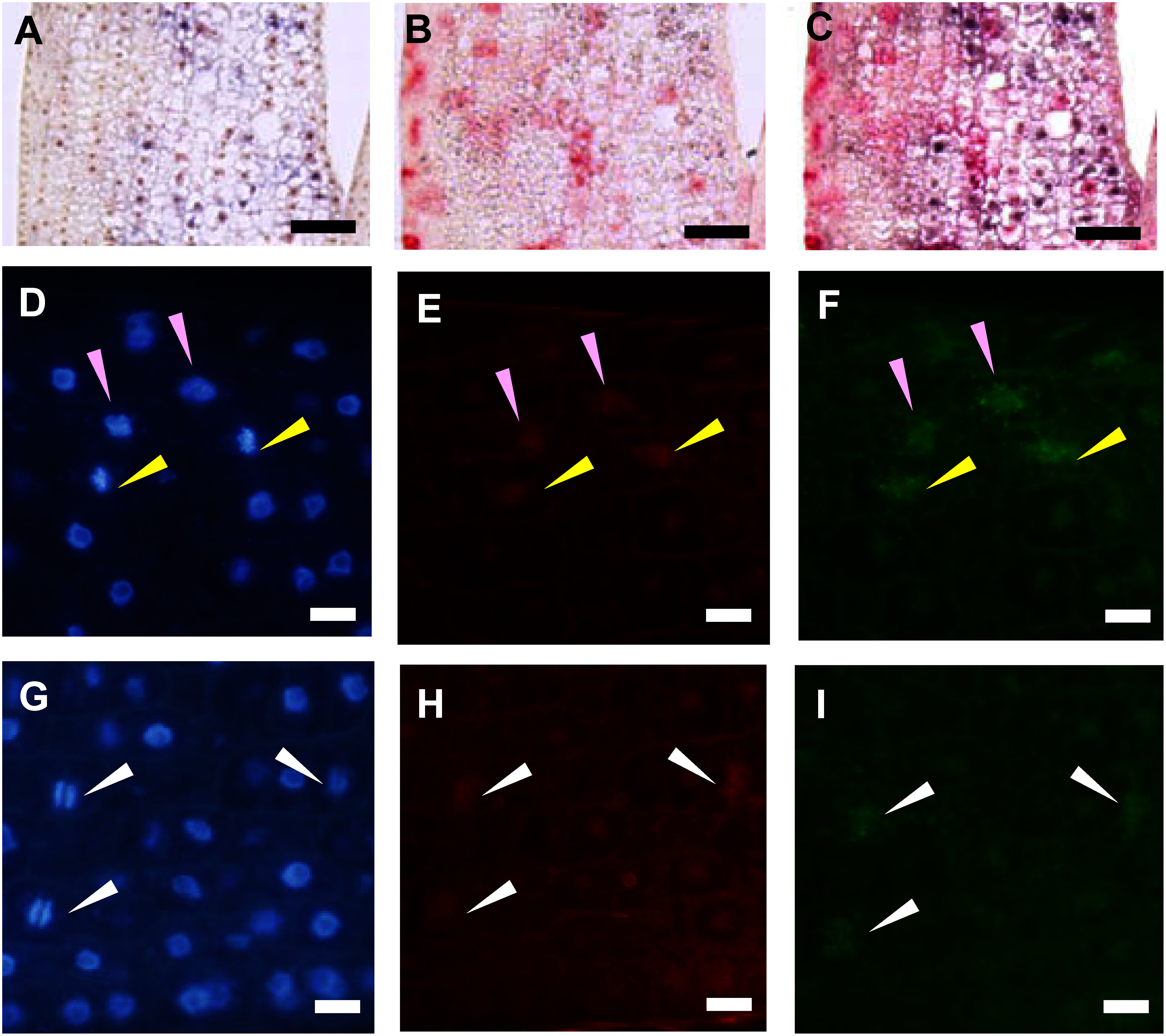
Figure 5. Double-stained rice leaf sheath with *KOR* and *Histone H4* (Panel A–C) and *KOR* and CycOs2 (Panels D–I). A: *KOR* expression pattern. B: *Histone H4* expression pattern. C: Overlayed image of A and B. D and G: DAPI staining. E and H: *KOR* expression pattern. F and I: *CycOs2* expression pattern. Panels A–C, D–F and G–I are the same section respectively. Pink, yellow and white arrowheads indicate prophase, metaphase and anaphase cells, respectively. The *KOR* signals do not overlap with the *Histone H4* ones, but they overlap with the *CycOs2* one. Bars: 50 µm in A–C, 10 µm in D–I.

To pinpoint the cell cycle stage when *KOR* functions, we conducted double targeted in situ hybridizations. First, we used *Histone H4* and *KOR* as probes revealing that the cells with a *Histone H4* signal did not coincide with the ones exhibiting a *KOR* signal and the frequency of *KOR* expressing cells were lower than that of *Histone H4* (Figure 5A–C). Next, we used *CycOs2*, which is expressed from the end of G2 phase to the end of M phase in rice (Umeda et al. 1999), as a probe for double staining with *KOR*. We found that the signal from these two genes overlapped (Figure 5D–I). Signals from both probes were observed in prophase, metaphase and anaphase cells. These results suggest that *KOR* is expressed during the M phase but not the S phase in the cell cycle.

Taken together, *KOR* plays a crucial role in proper cell division, presumably involving cytokinesis and the regular progression of the cell cycle. *KOR* is expressed during the M phase in the cells throughout the plant body, and dysfunction of the *KOR* gene induces a non-negligible number of cells with two nuclei, malformed cell shapes, and premature death. Further investigation of mature *kor* mutant plants revealed that KOR is also required for promoting the growth of leaves for a period after initiation through increased cell division, and ensuring the proper phase change from juvenile phase to adult phase during vegetative development in rice. Further investigations will shed light on the relationship between the cell-level function and the organ-level function of *KOR* gene and demonstrating how KOR functions pleiotropically in rice.

## Abbreviations

M phase: mitotic phase
PHP: phragmoplast
PPB: preprophase band
RAM: root apical meristem
S phase: synthetic phase
SAM: shoot apical meristem

## Description of Supplementary Files

Supplementary Figure S1. *KOR* expression data.
