# Supplementary material for: Rice KORPOKKUR gene is expressed in mitotic cells and regulates pleiotropic features during vegetative phase
Source: Plant Biotechnol (Tokyo). 2024 Jun 25;41(2):121–7. doi: 10.5511/plantbiotechnology.24.0305a (PMC11500571; doi:10.5511/plantbiotechnology.24.0305a)
Supplement: Supplementary Data [file plantbiotechnology-41-2-24.0305a-s001.pdf]

Supplementary Files

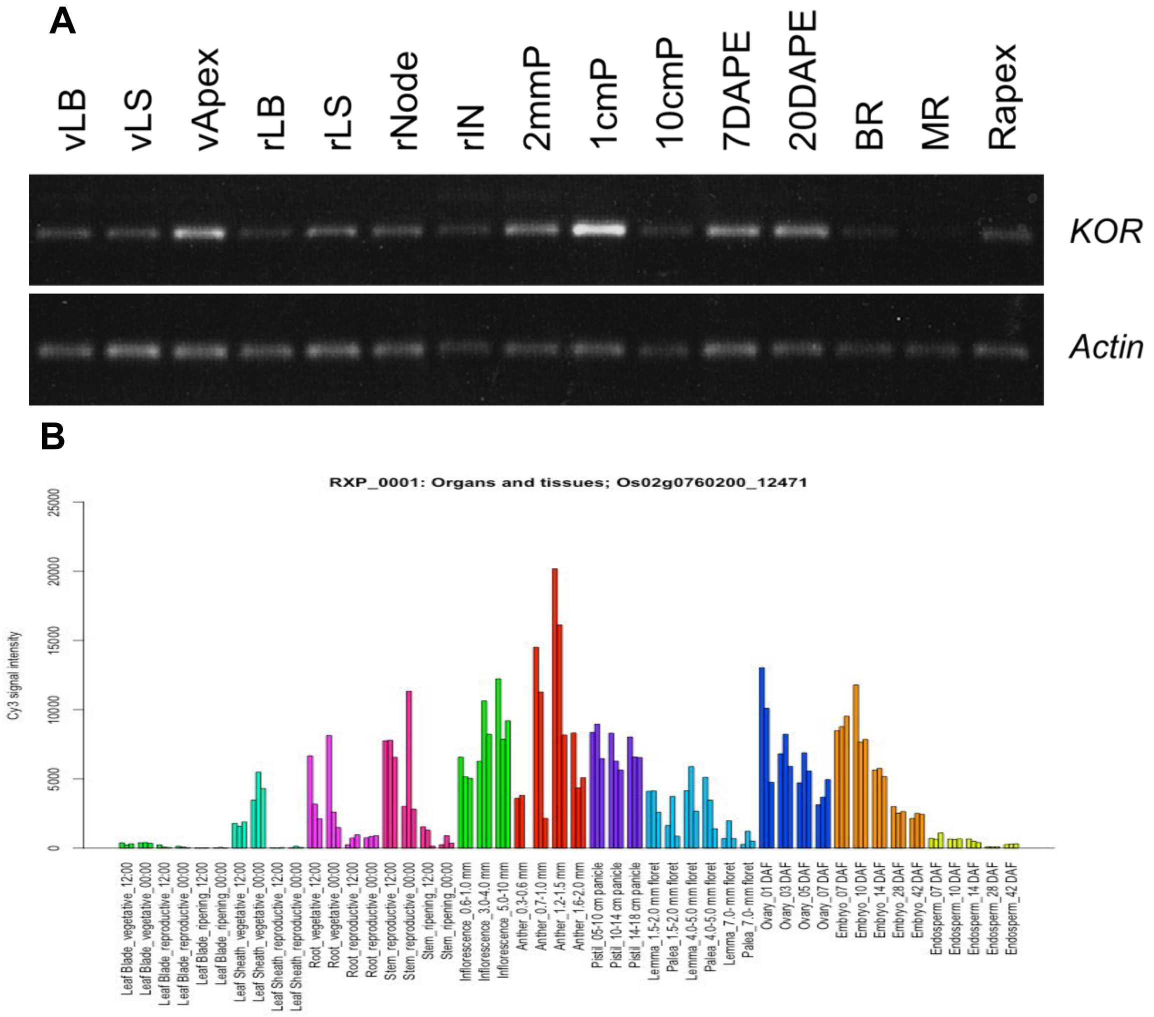

Supplementary Figure S1. *KOR* expression data.

A. Result from semi-quantitative PCR. *KOR* is expressed throughout the plant body with higher expression in the plant parts that include meristems.

   v; vegetative phase plants, r; reproductive phase plants, LB; leaf blade; LS; leaf sheath, IN; internode, P; immature panicle, DAP; days after pollination, E; embryo, BR; basal part of roots, MR; middle part of roots, Rapex; root apex.

B. Expression data from the Rice Gene Expression Profiling Database (RiceXpro).
